# Supplementary material for: NEMO reshapes the α-Synuclein aggregate interface and acts as an autophagy adapter by co-condensation with p62
Source: Nat Commun. 2023 Dec 19;14:8368. doi: 10.1038/s41467-023-44033-0 (PMC10730909; doi:10.1038/s41467-023-44033-0)
Supplement: Supplementary file 1 — Supplementary Information [file 41467_2023_44033_MOESM1_ESM.pdf]

a

Gene	Chromosome	GRCh37 position	Ref. allele	Alt. allele	Zygosity	Functional consequence	Transcript ID	cDNA change	Protein change	SNP ID	CADD score (v1.6)	gnomAD frequency	ACMG criteria	Pathogenicity	Phenotyp associated with variants in the respective gene	OMIM
VPS13D	1	12337667	C	T	heterozygous	missense variant	NM_015378	c.4022C>T	p.Ser1341Leu	rs12407578	24.6	0.0029	PM2, PP2, PP3	VUS	Autosomal recessive spinocerebellar ataxia 4 (SCAR4)	607317
PUM1	1	31438976	C	T	heterozygous	missense variant	NM_001020658	c.1939G>A	p.Gly647Ser	no SNP ID	25.3	0	PM2	VUS	Autosomal dominant spinocerebellar ataxia 47 (SCA47)	617931
SETX	9	135202325	A	C	heterozygous	missense variant	NM_015046	c.4660T>G	p.Cys1554Gly	rs112089123	25.0	0.0056	BS2, BP6	Likely benign	Autosomal dominant juvenile amyotrophic lateral sclerosis 4 and Autosomal recessive spinocerebellar ataxia with axonal neuropathy 2	602433 and 606002
VPS11	11	118948615	G	A	heterozygous	missense variant	NM_021729.6	c.1597G>A	p.Gly533Ser	rs782532693	22.8	1.219e-05	PM2, PP2, PP3	VUS	Autosomal recessive dystonia 31 and autosomal recessive hypomyelinating leukodystrophy 12	619637 and 616683
SCN8A	12	52168012	C	T	heterozygous	missense variant	NM_001330260.2	c.3685C>T	p.Arg1229Cys	rs773069251	25.5	0	PM2, PP2, PP3	VUS	Autosomal dominant familial myoclonus 2, autosomal dominant cognitive impairment with or without cerebellar ataxia, autosomal dominant developmental and epileptic encephalopathy 13, and autosomal dominant benign familial infantile seizures 5	618364, 614306, 614558, and 617080

GCCh37:Genome Reference Consortium Human Build 37;
SNP: single-nucleotide polymorphism;
CADD: Combined Annotation Dependent Depletion (PMID: 33618777)
gnomAD: The Genome Aggregation Database;
VUS: variant of uncertain significance
OMIM: Online Mendelian Inheritance in Man®

b

AARS1, AARS2, ABCB7, ABCD1, ABHD12, ACOX1, ADAR, ADCY5, AFG3L2, AIMP1, AIMP2, ALDH18A1, ALDH3A2, ALS2, ANG, ANO10, ANO3, AP4B1, AP4E1, AP4M1, AP4S1, AP5Z1, APP, APTX, ARL6IP1, ARSA, ASPA, ATL1, ATM, ATP13A2, ATP1A2, ATP1A3, ATP7B, ATP8A2, B4GALNT1, BCAP31, BOLA3, BSCL2, C12orf65, C19orf12, CA2, CA8, CACNA1A, CACNA1G, CACNB4, CAMTA1, CAPN1, CARS2, CCDC88C, CHCHD10, CHCHD2, CHMP2B, CIZ1, CLCN2, CLN3, CLN5, CLN6, CLN8, COA7, COASY, COL4A1, COL4A2, COL6A3, COLGALT1, COQ8A, COX10, COX15, COX20, COX6B1, CP, CPT1C, CSF1R, CTBP1, CTC1, CTSA, CTSD, CTSF, CWF19L1, CYP27A1, CYP2U1, CYP7B1, DARS1, DARS2, DCAF17, DCTN1, DDHD1, DDHD2, DNAJC12, DNAJC5, DNAJC6, DNMT1, DSTYK, EARS2, EIF2B1, EIF2B2, EIF2B3, EIF2B4, EIF2B5, ELOVL4, ELOVL5, EMC1, EPRS1, ERCC6, ERCC8, ERLIN2, FA2H, FAM126A, FARS2, FBXO7, FGF14, FIG4, FKRP, FKTN, FLVCR1, FOLR1, FOXC1, FOXRED1, FRRS1L, FTL, FUCA1, FUS, FXN, GALC, GAN, GBA, GBA2, GBE1, GCDH, GCH1, GEMIN4, GFM1, GJC2, GLA, GLB1, GLRX5, GMPPB, GNAL, GNAO1, GOSR2, GRID2, GRM1, GRN, HEPACAM, HEXA, HEXB, HIKESHI, HNRNPA1, HPCA, HSD17B4, HSPD1, HTRA1, IBA57, IDS, IFIH1, IRF2BPL, ISCA1, ISCA2, ITM2B, ITPR1, KARS1, KCNA1, KCNC3, KCND3, KCNJ10, KCNMA1, KCNT1, KCTD7, KIDINS220, KIF1A, KIF1C, KIF5A, KMT2B, L1CAM, L2HGDH, LAMA2, LARGE1, LIPT2, LMNB1, LRPPRC, LRRK2, LYRM7, MAG, MAPT, MARS2, MATR3, MCOLN1, MECR, MFSD8, MLC1, MPV17, MRE11, MTFMT, MTHFS, MTTP, MYORG, NACC1, NARS2, NAXE, NDUFS1, NDUFV1, NFU1, NIPA1, NKX6-2, NOTCH3, NPC1, NPC2, NT5C2, NUBPL, OCLN, OPTN, PANK2, PARK7, PC, PDE10A, PDGFB, PDGFRB, PET100, PEX1, PEX10, PEX12, PEX13, PEX14, PEX16, PEX19, PEX2, PEX26, PEX3, PEX5, PEX6, PEX7, PFN1, PHGDH, PHYH, PINK1, PLA2G6, PLAA, PLEKHG2, PLP1, PMPCA, PMPCB, PNKD, PNKP, PNPLA6, POLG, POLR3A, POLR3B, POLR3K,POMGNT1, POMT1, POMT2, PPT1, PRICKLE1, PRKCG, PRKN, PRKRA, PRNP, PRRT2, PSAP, PSAT1, PSEN1, PSEN2, PUM1, PYCR2, QARS1, RAB11B, RARS1, RARS2, REEP1, REEP2, RNASEH2A, RNASEH2B, RNASEH2C, RNASET2, RNF170, RNF216, RTN2, SACS, SAMHD1, SCN1A, SCN2A, SCN8A, SCP2, SCYL1, SDHA, SDHAF1, SETX, SGCE, SIGMAR1, SIL1, SLC16A2, SLC17A5, SLC19A3, SLC1A3, SLC1A4, SLC20A2, SLC25A12, SLC2A1, SLC30A10, SLC33A1, SLC39A14, SLC6A3, SNCA, SNX14, SOD1, SOX10, SPART, SPAST, SPG11, SPG21, SPG7, SPR, SPTAN1, SPTBN2, SQSTM1, STN1, STUB1, SUMF1, SURF1, SYNE1, SYNJ1, TACO1, TARDBP, TARS2, TBCD, TBCK, TBK1, TDP1, TDP2, TECPR2, TFG, TGFB1, TGM6, TH, THAP1, TMEM106B, TMEM240, TOR1A, TPP1, TRAPPC12, TRAPPC6B, TRAPPC9, TREX1, TTBK2, TTC19, TTPA, TUBA4A, TUBB4A, TUFM, TWNK, TYMP, TYROBP, UBAP1, UBTF, UCHL1, UFM1, VAC14, VAMP1, VAPB, VARS1, VARS2, VCP, VLDLR, VPS11, VPS13A, VPS13C, VPS13D, VPS35, WARS2, WASHC5, WDR45, WDR45B, WDR81, WFS1, WWOX, XK, XPR1, XRCC1, ZFYVE26, ZNHIT3

Supplementary Figure 1. Whole-genome sequencing of the patient harbouring the p.Q330* NEMO mutation.

a Upon whole-genome sequencing of the DNA extracted from the primary skin fibroblasts from our patient, the presence of coding or splice-site variants in 380 genes associated with neurodegenerative disorders (b) were analyzed in detail. 508 exonic and splice site variants were identified in these genes. After filtering out 316 synonymous single-nucleotide variants, 192 candidate changes were left. In the next step, the list of candidate variants was narrowed to only 16 variants found with frequency below 1% in the Genome Aggregation Database (gnomAD). After excluding the 10 variants with Combined Annotation Dependent Depletion (CADD) score below 20, five heterozygous missense variants in the *VPS13D, PUM1, SGCE, SETX, VPS11*, and *SCN8A* genes remained, which are likely begin (*SETX*) or of uncertain significance.

b List of analyzed genes.

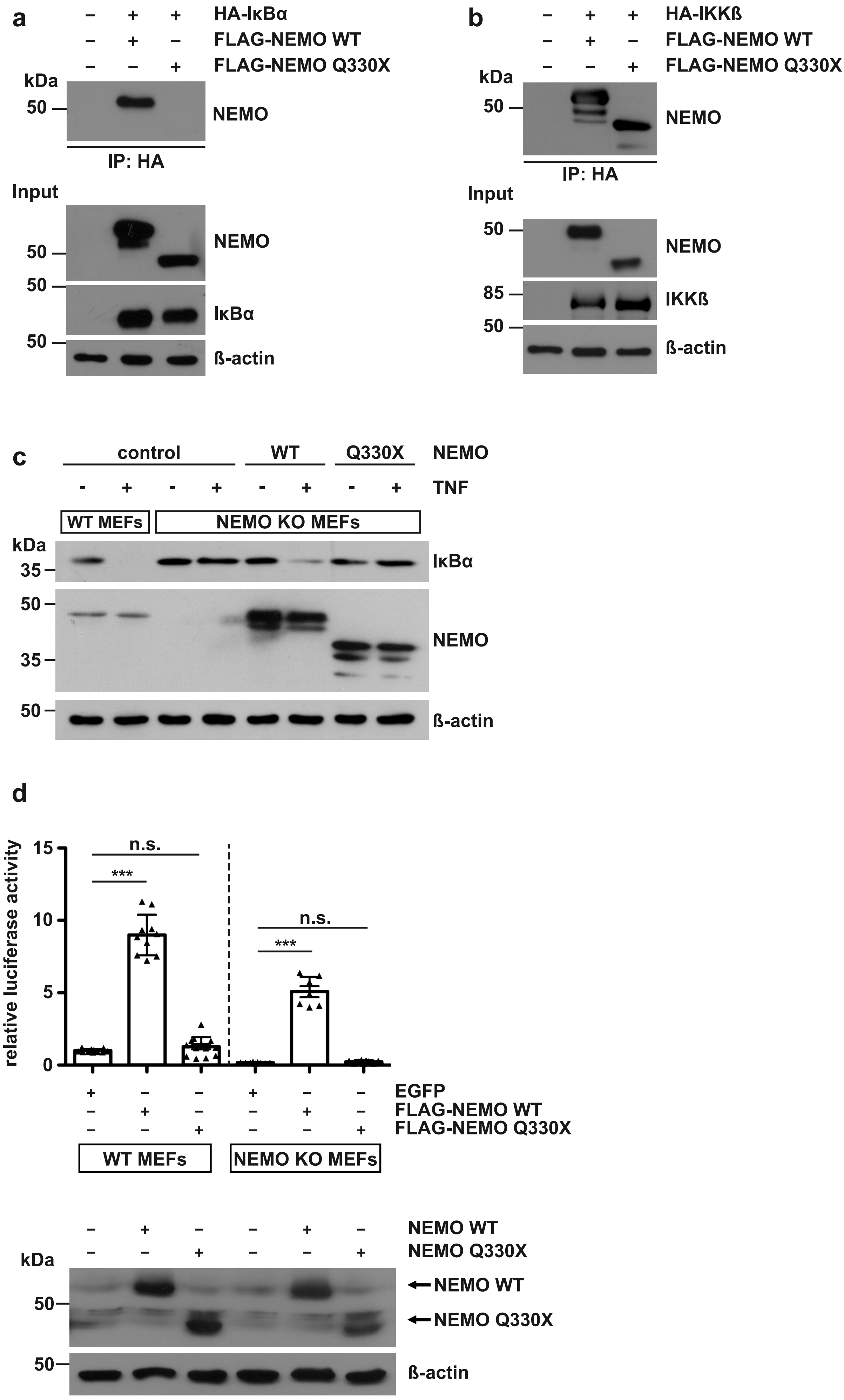

a
HA-IκBα
FLAG-NEMO WT
FLAG-NEMO Q330X
kDa
50
NEMO
IP: HA
Input
NEMO
50
50
IκBα
50
ß-actin
b
HA-IKKß
FLAG-NEMO WT
FLAG-NEMO Q330X
kDa
50
NEMO
IP: HA
Input
50
NEMO
85
IKKß
50
ß-actin
c
control
WT
Q330X
NEMO
TNF
WT MEFs
NEMO KO MEFs
kDa
35
IκBα
50
35
NEMO
50
ß-actin
d
relative luciferase activity
15
10
5
0
n.s.
***
n.s.
***
EGFP
FLAG-NEMO WT
FLAG-NEMO Q330X
WT MEFs
NEMO KO MEFs
NEMO WT
NEMO Q330X
kDa
50
NEMO WT
NEMO Q330X
50
ß-actin

Supplementary Figure 2. The Q330X NEMO mutant is defective in NF-κB signaling.

a, b The Q330X mutation disrupts binding of NEMO to IκBα but not to IKKβ. HEK293T cells were transiently transfected with wildtype (WT) FLAG-NEMO or Q330X FLAG-NEMO and HA-IκBα (a) or HA-IKKβ (b) as indicated. One day after transfection, the cells were lysed and HA-tagged proteins were immunoprecipitated using anti-HA-beads followed by immunoblotting using antibodies against NEMO. The input was immunoblotted for NEMO, IκBα (a) or IKKβ (b) and β-actin.

c Wildtype NEMO but not Q330X NEMO rescues defective IκBα degradation in NEMO KO MEFs. WT and NEMO KO MEFs were transiently transfected with WT FLAG-NEMO, Q330X FLAG-NEMO or luciferase as a control. One day after transfection, the cells were treated with TNF (25 ng/ml, 15 min) as indicated or left untreated and analyzed by immunoblotting using antibodies against IκBα, NEMO and β-actin.

d In contrast to WT NEMO, Q330X NEMO does not promote NF-κB transcriptional activity. WT and NEMO KO MEFs were transiently transfected with an NF-κB luciferase reporter plasmid and WT FLAG-NEMO, Q330X FLAG-NEMO, or EGFP as a control. 24 h after transfection, the cells were lysed and luciferase activity was measured luminometrically using a plate reader. Data represent the means ± SD of three independent experiments consisting of at least three technical replicates each. n= 15/10/13 (wt), 9/7/9 (NEMO KO), Statistics: Student's t-test. ***$p \leq 0.001$. Lower panel: Cell lysates were immunoblotted using antibodies against NEMO and β-actin (input control).

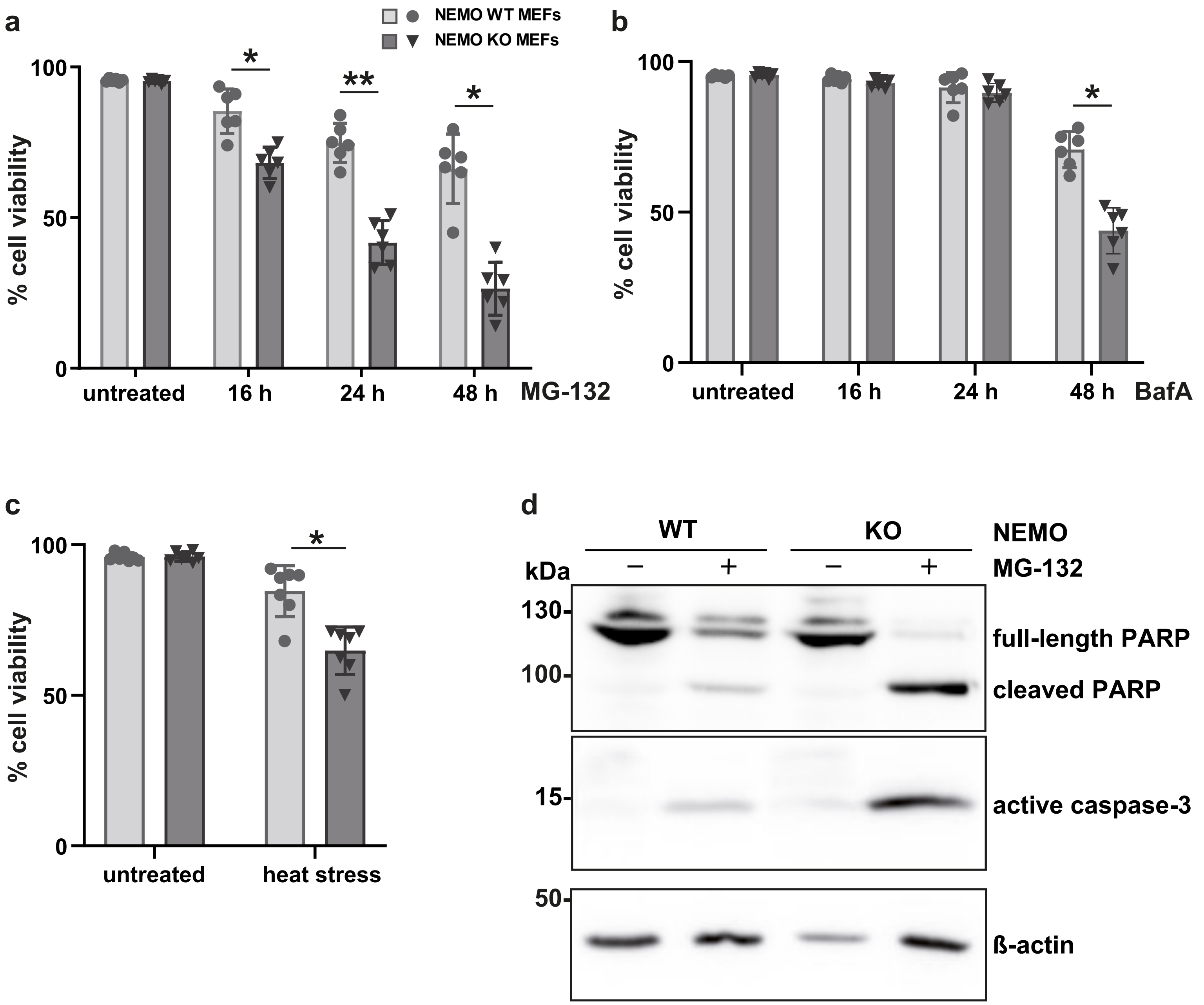
a
NEMO WT MEFs
NEMO KO MEFs
% cell viability
100
50
0
*
**
*
untreated
16 h
24 h
48 h
MG-132
b
% cell viability
100
50
0
*
untreated
16 h
24 h
48 h
BafA
c
% cell viability
100
50
0
*
untreated
heat stress
d
WT
KO
NEMO
kDa
−
+
−
+
MG-132
130
100
full-length PARP
cleaved PARP
15
active caspase-3
50
ß-actin

Supplementary Figure 3. NEMO KO MEFs are vulnerable to proteotoxic stress.

a, b NEMO deficiency decreases cell viability upon proteasomal or autophagosomal inhibition. WT and NEMO KO MEFS were treated with MG-132 for 16 h (2 µM), 24 h (2 µM), or 48 h (0.5 µM), or with Bafilomycin A1 (BafA) for 16 h (500 nM), or 24 h (500 nM), or 48 h (100 nM) or left untreated. Cell viability was quantified using Trypan blue exclusion. Data are displayed as mean ± SD and were analyzed by two-way ANOVA followed by Tukey's multiple comparisons test, n= 6 individual experiments. *p ≤ 0.05, **p ≤ 0.01.

c NEMO deficiency decreases cell viability upon heat stress. WT and NEMO KO MEFs were subjected to heat stress (46°C, 1 h), followed by overnight recovery. Cell viability was quantified using Trypan blue exclusion. Data are displayed as mean ± SD and were analyzed by two-way ANOVA followed by Bonferroni´s multiple comparison test, n= 7 individual experiments. *p ≤ 0.05.

d NEMO deficiency increases apoptotic cell death upon proteasomal inhibition. WT and NEMO KO MEFS were treated with MG-132 (2 µM, 24 h) or left untreated. Cell lysates were then analyzed by immunoblotting using antibodies against PARP, active caspase-3, and β-actin.

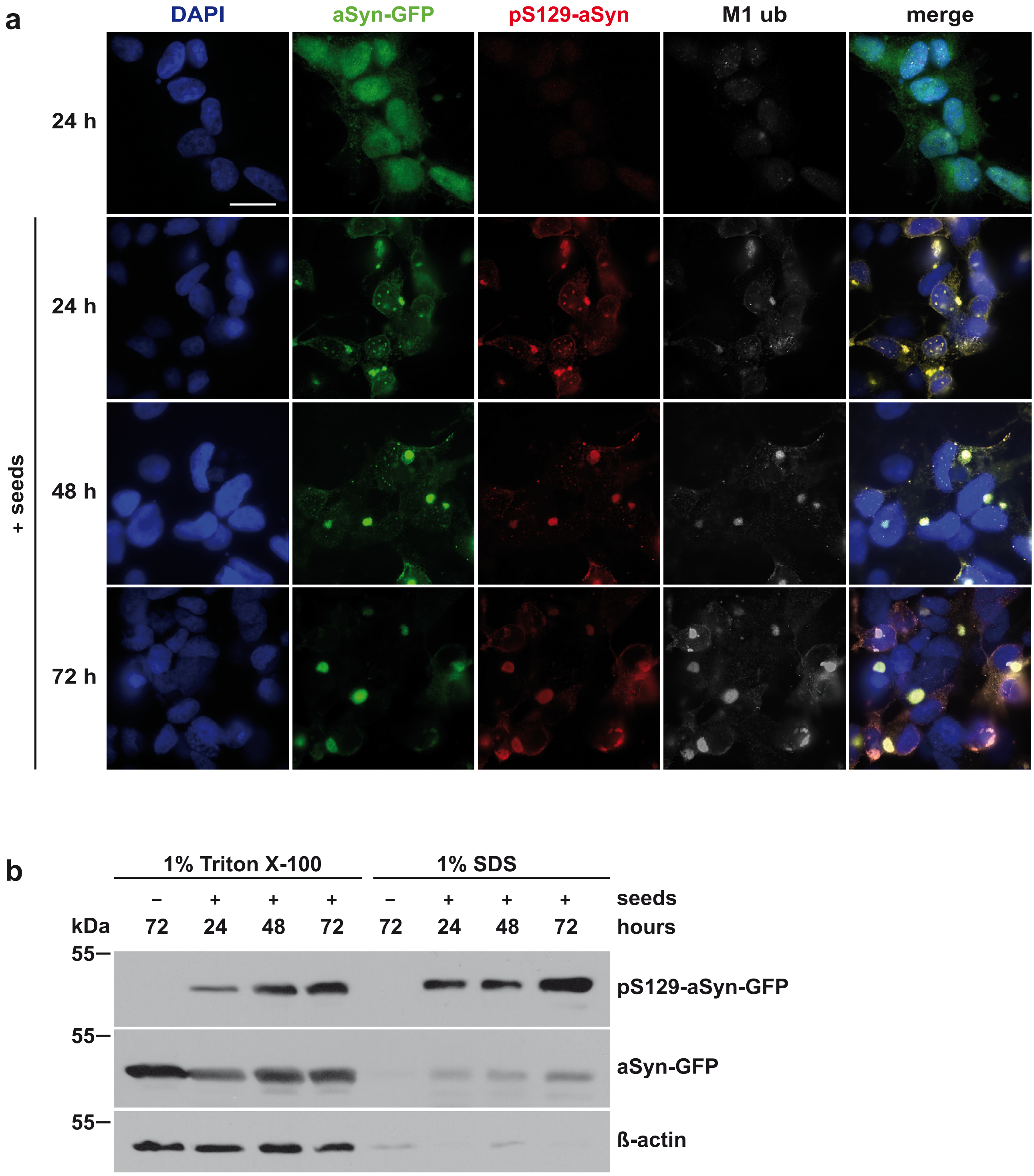
a
DAPI
aSyn-GFP
pS129-aSyn
M1 ub
merge
24 h
+ seeds
24 h
48 h
72 h
b
1% Triton X-100
1% SDS
– + + + – + + + seeds
kDa 72 24 48 72 72 24 48 72 hours
55
pS129-aSyn-GFP
55
aSyn-GFP
55
ß-actin

Supplementary Figure 4. aSyn A53T seeds induce aggregation of pS129-positive aSyn-GFP aggregates that colocalize with M1-linked ubiquitin.

a aSyn-GFP aggregates are phosphorylated at S129 and are decorated by M1-linked ubiquitin. SH-SY5Y cells stably expressing aSyn A53T-GFP were treated with aSyn A53T seeds (+ seeds) or PBS as a control, fixed 24 h, 48 h, or 72 h after seeding, and analyzed by immunocytochemistry and fluorescence SR-SIM using antibodies against pS129-aSyn and M1-linked ubiquitin. Scale bar, 20 µm.

b aSyn A53T seeds induce aggregation and phosphorylation of aSyn-GFP at S129. SH-SY5Y cells stably expressing aSyn A53T-GFP were treated with aSyn A53T seeds or PBS as a control and harvested 24 h, 48 h, or 72 h after seeding. The cells were subjected to a detergent solubility assay and analyzed by immunoblotting using antibodies against pS129-aSyn, aSyn, and β-actin.

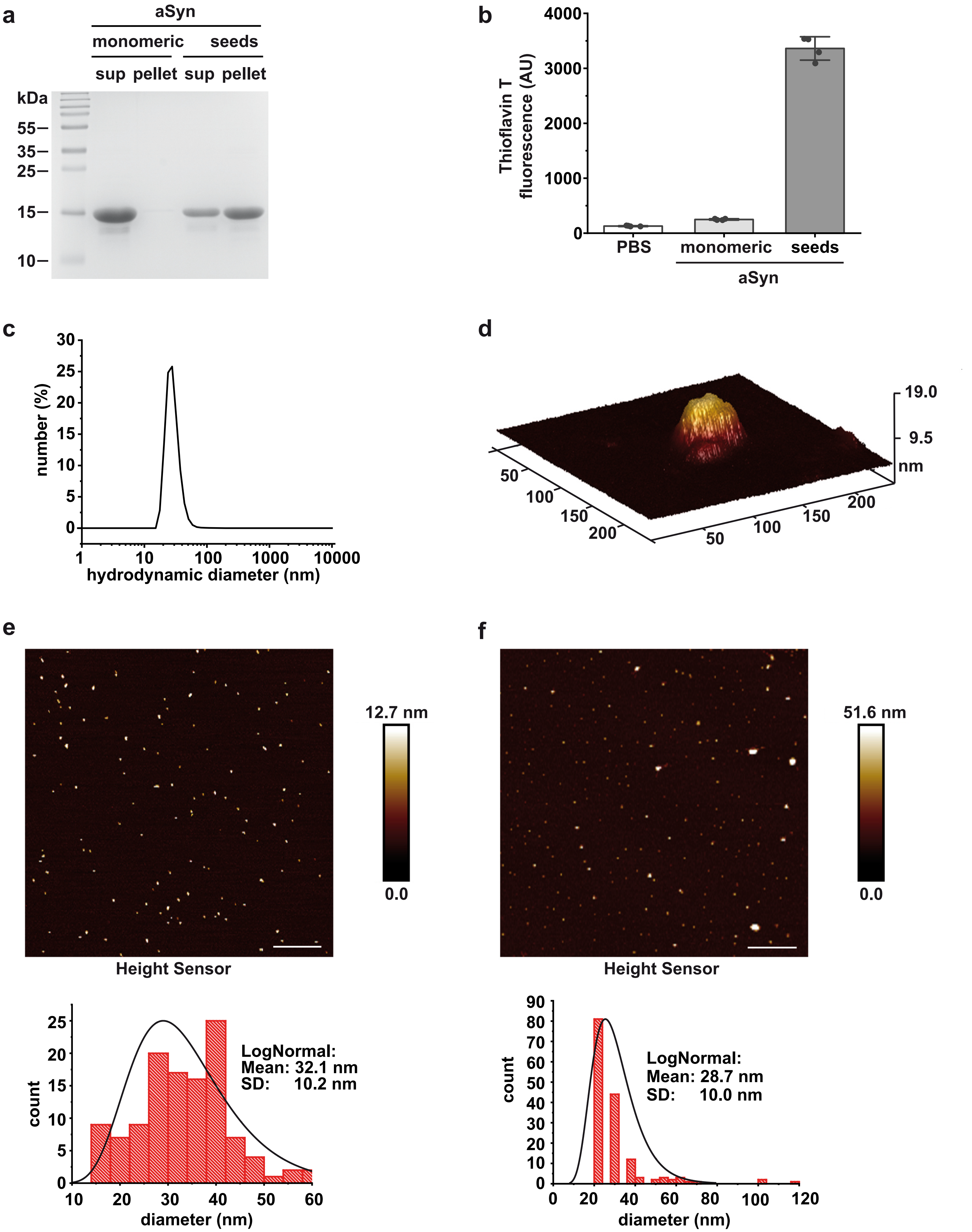
a
aSyn
monomeric
seeds
sup
pellet
sup
pellet
kDa
55
35
25
15
10
b
Thioflavin T fluorescence (AU)
4000
3000
2000
1000
0
PBS
monomeric
seeds
aSyn
c
number (%)
30
25
20
15
10
5
0
1
10
100
1000
10000
hydrodynamic diameter (nm)
d
19.0
9.5
nm
50
100
150
200
50
100
150
200
e
12.7 nm
0.0
Height Sensor
count
diameter (nm)
LogNormal:
Mean: 32.1 nm
SD: 10.2 nm
f
51.6 nm
0.0
Height Sensor
count
diameter (nm)
LogNormal:
Mean: 28.7 nm
SD: 10.0 nm

Supplementary Figure 5. Validation of aSyn A53T seeds.

a Sedimentation assay for monomeric aSyn A53T and aSyn A53T seeds. *In vitro* formed aSyn A53T seeds were separated from soluble aSyn A53T monomers by centrifugation. Supernatant (sup) and pellet fractions were analyzed by SDS-PAGE and Coomassie staining.

b aSyn A53T seeds bind to Thioflavin T. Fluorometry of monomeric aSyn, aSyn seeds or PBS as a control was performed using a microplate reader. Data are shown as mean ± SD, n= 4 technical replicates for each sample.

c Derived hydrodynamic diameter of aSyn A53T seeds measured by dynamic light scattering (DLS). The size distribution of freshly sonicated aSyn A53T seeds dispersed in PBS was evaluated from the derived intensity, considering the number distribution of colloids. A mean diameter of 27.7 nm with a standard deviation of 5.7 nm was derived from a Gaussian fit.

d Analysis of aSyn A53T seeds by atomic force microscopy (AFM). 3D rendered image of a single aSyn A53T seed adsorbed on MICA from a freshly sonicated dispersion of aSyn A53T seeds in PBS, measured by liquid AFM in peak force mode. Rendering was done on a 240 x 240 nm height sensor image by NanoScope Analysis software from Bruker. The measured feature exhibits a vertical length of 59 nm, a horizontal length of 41 nm and a maximum height of 10 nm.

e aSyn size distribution of LAFM height measurements on MICA in PBS solution. Obtained 2D mapping of the measured surface (Top: size 6.6 x 6.6 μm). Particle size distribution was derived by particle analysis mode of the Bruker Nanoscope software: Height threshold was set to > 5 nm. Statistical distribution was evaluated with a Log normal function: Mean particle diameter 32.1 ± 10.2 nm.

f aSyn size distribution of AFM height measurements on Si-wafer after 4 μl dropcasting. Obtained 2D mapping of the measured surface (Top: size 6.6 x 6.6 μm). Particle size distribution was derived by particle analysis mode of the Bruker Nanoscope software: Height threshold was set to > 16 nm. Statistical distribution was evaluated with a Lognormal function: Mean particle diameter 28.7 ± 10.0 nm.

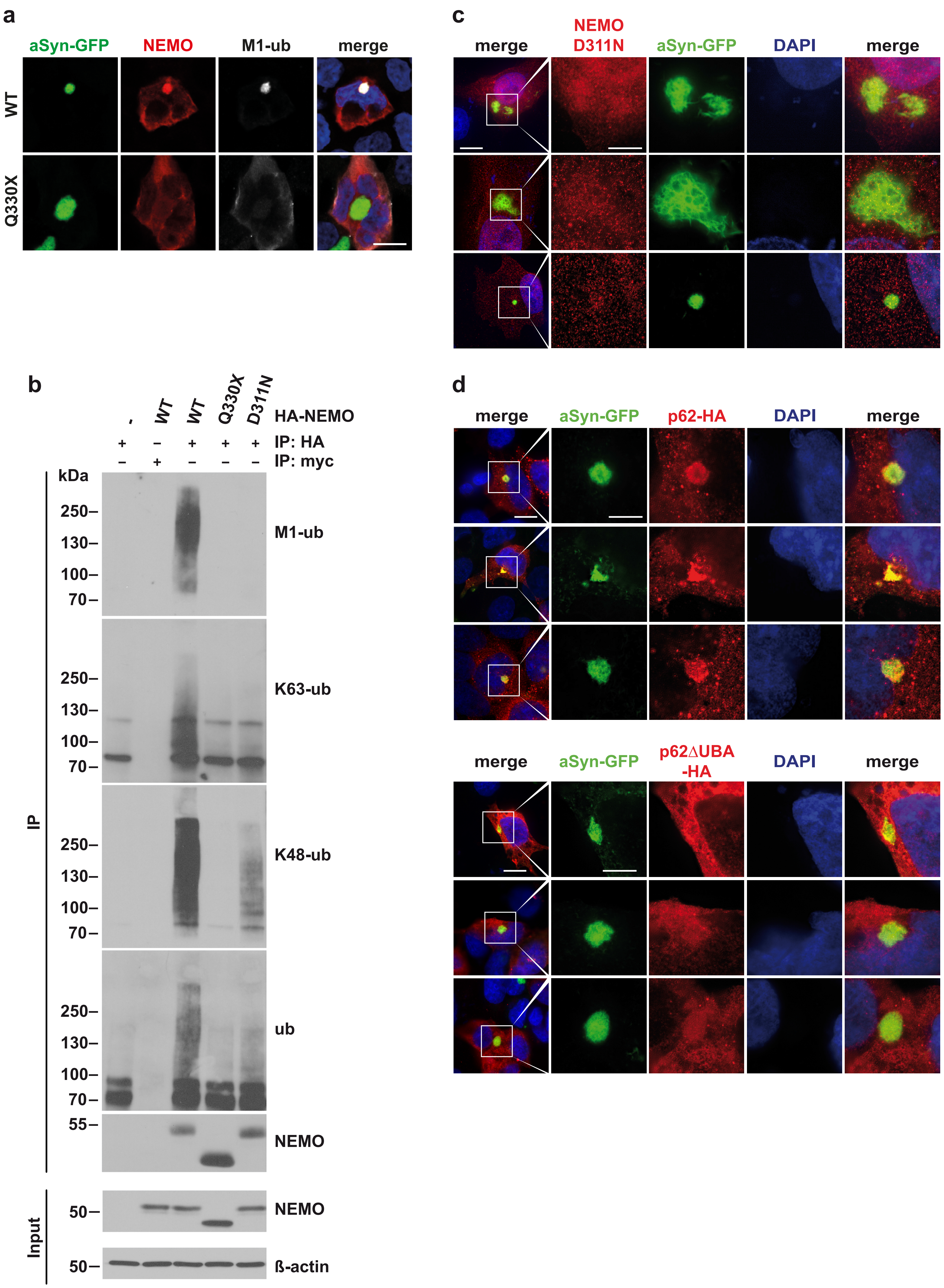
a
aSyn-GFP
NEMO
M1-ub
merge
WT
Q330X
b
-
WT
WT
Q330X
D311N
HA-NEMO
+ – + + +
IP: HA
– + – – –
IP: myc
kDa
250–
130–
100–
70–
M1-ub
250–
130–
100–
70–
K63-ub
250–
130–
100–
70–
K48-ub
250–
130–
100–
70–
ub
55–
NEMO
IP
50–
NEMO
50–
ß-actin
Input
c
merge
NEMO D311N
aSyn-GFP
DAPI
merge
d
merge
aSyn-GFP
p62-HA
DAPI
merge
merge
aSyn-GFP
p62ΔUBA -HA
DAPI
merge

Supplementary Figure 6. Impaired recruitment of mutant NEMO and p62ΔUBA to aSyn aggregates.

a The abundance of M1-linked ubiquitin chains at aSyn aggregates is increased by WT but not Q330X NEMO. CRISPR/Cas9 NEMO KO SH-SY5Y cells were transiently transfected with aSyn A53T-GFP and either WT FLAG-NEMO or Q330X FLAG-NEMO. One day after transfection, the cells were treated with aSyn A53T seeds, fixed 48 h after seeding and analyzed by immunohistochemistry and fluorescence SR-SIM using anti-antibodies against M1-ubiquitin and FLAG-Tag. Scale bar, 10 µm.

b Modification of Q330X and D331N NEMO with ubiquitin is impaired. HEK293T cells were transiently transfected with plasmids encoding HA-tagged wildtype NEMO, Q330X NEMO, or D311N NEMO. Cells were lysed under denaturing conditions and the HA-tag was immunoprecipitated using anti-HA agarose; anti-myc-agarose served as a control for unspecific binding. Samples were analyzed by immunoblotting with antibodies specific for M1-linked, K63-linked, or K48-linked ubiquitin, or pan-ubiquitin.

c D311N NEMO is not recruited to aSyn aggregates. SH-SY5Y cells stably expressing aSyn A53T-GFP were transiently transfected with D311N HA-NEMO. After 24 h, the cells were treated with aSyn A53T seeds, fixed 48 h after seeding, and analyzed by immunocytochemistry and fluorescence SR-SIM using an antibody against the HA-tag. Scale bar, 10 µm (overview) and 5 µm (inset).

d Recruitment of p62 lacking the UBA domain aSyn aggregates is impaired. SH-SY5Y cells stably expressing aSyn A53T-GFP were transiently transfected with plasmids encoding either HA-tagged p62 or p62ΔUBA as indicated. After 24 h, the cells were treated with aSyn A53T seeds, fixed 48 h later, and analyzed by immunocytochemistry and fluorescence SR-SIM using an antibody against the HA-tag. Scale bar, 10 µm (overview) and 5 µm (inset).

Pathology	Brain region	Identifier	Type	source
PD	midbrain	T92/174	Paraffin	Charité, Berlin, Germany
AD	frontal isocortex	A133/17	Paraffin	Institute of Neuropathology, University Medical Center, Hamburg-Eppendorf, Germany
FTLD	frontal isocortex	199	Paraffin	
DLBD	frontal isocortex	A199/17, A206/16	Paraffin	
NEMO patient	middle frontal gyrus	A16-153	Paraffin	Department of Pathology, University of California, San Francisco, California, USA.
DLBD	middle frontal gyrus	A17-159	Paraffin	
control	middle frontal gyrus	A17-47	Paraffin	

Supplementary Table 1. ***Post mortem*** **brain samples data**
